# Polyplex Nanomicelle‐Mediated *Pgc‐1α4* mRNA Delivery Via Hydrodynamic Limb Vein Injection Enhances Damage Resistance in Duchenne Muscular Dystrophy Mice

**DOI:** 10.1002/advs.202409065

**Published:** 2025-03-06

**Authors:** Xuan Du, Hideyuki Nakanishi, Takashi Yamada, Yooksil Sin, Katsura Minegishi, Norio Motohashi, Yoshitsugu Aoki, Keiji Itaka

**Affiliations:** ^1^ Department of Biofunction Research Laboratory for Biomaterials and Bioengineering, Institute of Integrated Research Institute of Science Tokyo Tokyo 101‐0062 Japan; ^2^ Clinical Biotechnology Team Center for Infectious Disease Education and Research (CiDER) Osaka University Osaka 565‐0871 Japan; ^3^ Department of Physical Therapy Sapporo Medical University Sapporo 060‐8556 Japan; ^4^ Department of Molecular Therapy National Institute of Neuroscience National Center of Neurology and Psychiatry (NCNP) Tokyo 187‐8502 Japan

**Keywords:** duchenne muscular dystrophy (DMD), lipid nanoparticle (LNP), mRNA therapeutics, peroxisome proliferator‐activated receptor gamma coactivator (PGC‐1α), polyplex nanomicelle

## Abstract

Duchenne muscular dystrophy (DMD) is caused by mutations in the *DMD* gene, leading to the absence of dystrophin and progressive muscle degeneration. Current therapeutic strategies, such as exon‐skipping and gene therapy, face limitations including truncated dystrophin production and safety concerns. To address these issues, a novel mRNA‐based therapy is explored using polyplex nanomicelles to deliver mRNA encoding peroxisome proliferator‐activated receptor gamma coactivator 1 alpha isoform 4 (PGC‐1α4) via hydrodynamic limb vein (HLV) administration. Using an in vivo muscle torque measurement technique, it is observed that nanomicelle‐delivered *Pgc‐1α4* mRNA significantly improved muscle damage resistance and mitochondrial activity in mdx mice. Specifically, HLV administration of *Pgc‐1α4* mRNA in dystrophic muscles significantly relieved the torque reduction and myofiber injury induced by eccentric contraction (ECC), boosted metabolic gene expression, and enhanced muscle oxidative capacity. In comparison, lipid nanoparticles (LNPs), a widely used mRNA delivery system, does not achieve similar protective effects, likely due to their intrinsic immunogenicity. This foundational proof‐of‐concept study highlights the potential of mRNA‐based therapeutics for the treatment of neuromuscular diseases such as DMD and demonstrates the capability of polyplex nanomicelles as a safe and efficient mRNA delivery system for therapeutic applications.

## Graphical abstract

This study presents a novel approach for treating Duchenne muscular dystrophy using mRNA encoding PGC‐1α4. Nanomicelle‐delivered Pgc‐1α4 mRNA enhances muscle damage resistance and mitochondrial activity in dystrophic muscles. This study demonstrates the potential of mRNA therapy for neuromuscular diseases like Duchenne muscular dystrophy and highlights polyplex nanomicelles as a safe and efficient mRNA delivery system for therapeutic applications.

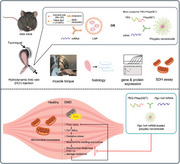

## 1 Introduction

Duchenne muscular dystrophy (DMD) is a genetic disorder caused by mutations in the *DMD* gene encoding dystrophin, a 427 kDa protein essential for maintaining the muscle fiber structure.^[^ ^1^ ^]^ The absence of dystrophin leads to progressive muscle wasting and early mortality. While there is no cure for DMD, strategies such as exon‐skipping, stop‐codon read‐through, and vector‐mediated gene therapy have made clinical progress.^[^ ^1^, ^2^ ^]^ To date, four exon‐skipping drugs (Eteplirsen, Golodirsen, Vitolarsen, and Casimersen) and one stop‐codon read‐through drug (Ataluren) have been approved by the U.S. Food and Drug Administration (FDA) or the European Medicines Agency (EMA). However, these drugs have shown limited efficacy in dystrophin restoration (less than 5%),^[^ ^2^ ^]^ and their mutation‐specific nature limits the applicability across all patients. Vector‐mediated gene therapy, on the other hand, is expected to be applicable to a wider range of patients. The recent approval of Elevidys by the FDA marks it as the first gene therapy approved for the treatment of DMD.^[^ ^3^ ^]^ Elevidys employs adeno‐associated virus (AAV) vectors to deliver micro‐dystrophin, a 138 kDa protein that encapsulates the functional domains of dystrophin. Nonetheless, this approach's reliance on AAV vectors introduces challenges, such as immune responses and liver toxicity,^[^ ^4^, ^5^, ^6^ ^]^ which may hinder the viability of repeated administrations.

Given these limitations of existing treatment options, there is an imperative need for innovative therapeutic strategies that can treat DMD regardless of mutation type while also minimizing safety concerns associated with delivery mechanisms. Messenger RNA (mRNA) therapy emerges as a compelling candidate. By directly harnessing the cell's protein synthesis machinery, this approach eliminates the need for viral vectors and thus reduces the associated toxicity and immune responses that complicate repeated administrations.^[^ ^7^, ^8^, ^9^ ^]^ Furthermore, as a versatile therapeutic modality, mRNA therapy allows for rapidly designing and synthesizing mRNAs that encode any protein of interest. This provides a straightforward connection between pathological analysis and treatment, potentially directly replenishing the deficient or aberrant proteins underlying DMD pathology.

The challenge of using mRNA for therapeutic purposes lies in the inflammatory response that occurs at the site of administration. The success of mRNA vaccines for COVID‐19 partly depended on the ability of lipid nanoparticles (LNPs) to efficiently induce immunity.^[^ ^10^ ^]^ However, unlike their application in vaccines, administering therapeutic mRNA using LNPs may not effectively achieve beneficial outcomes due to the toxicity and induced immune response.^[^ ^11^, ^12^, ^13^, ^14^ ^]^ In this study, we propose polyplex nanomicelle as an alternative mRNA carrier that allows for administration with reduced inflammatory response. Formed by the self‐assembly of mRNA and a synthetic block‐copolymer composed of polyethylene glycol (PEG) and polyamino acid (Poly{N‐[N′‐(2‐aminoethyl)‐2‐aminoethyl]aspartamide}) (PEG‐PAsp(DET)),^[^ ^15^ ^]^ the nanomicelles have a uniform diameter of ≈50 nm (see Table S1, Supporting Information) and feature a core‐shell structure with a dense PEG palisade on the surface (detailed in Figure 3A). Our recent comparative study between LNPs and nanomicelles for mRNA delivery into the muscle showed that nanomicelles provided less but sustained protein production, with significantly reduced immune responses and tissue damage.^[^ ^16^ ^]^

For DMD treatment, we selected peroxisome proliferator‐activated receptor gamma coactivator‐1 alpha (PGC‐1α), a promising therapeutic target among various dysregulated proteins in DMD.^[^ ^17^, ^18^ ^]^ As a master regulator of mitochondria, PGC‐1α is critical for mitochondrial biogenesis and energy metabolism in the skeletal muscle^[^ ^19^, ^20^, ^21^, ^22^, ^23^, ^24^, ^25^ ^]^ and has been reported to interact with a broad range of genes.^[^ ^26^, ^27^, ^28^, ^29^, ^30^, ^31^, ^32^, ^33^ ^]^ Indeed, overexpression of PGC‐1α in rodent models of DMD has yielded beneficial outcomes in various aspects, such as mitochondrial function, neuromuscular junction (NMJ) regulation, and damage resistance,^[^ ^34^, ^35^, ^36^, ^37^, ^38^, ^39^, ^40^ ^]^ although the mechanisms underlying such improvements remain elusive. In addition, Ruas et al., first identified PGC‐1α4 as a shorter isoform of the previously described PGC‐1α (hereafter referred to as PGC‐1α1) abundantly expressed in skeletal muscle upon exercise.^[^ ^41^ ^]^ PGC‐1α4 has been found to promote muscle hypertrophy by upregulating *Igf1*,^[^ ^41^ ^]^ enhance anaerobic glycolysis via *Glut4*,^[^ ^42^ ^]^ and promote angiogenesis via *Vegfa*.^[^ ^43^ ^]^ Also, AAV‐mediated muscle‐specific PGC‐1α4 delivery alleviated aging‐related muscle weakness.^[^ ^44^ ^]^ These findings led us to select PGC‐1α4 as a treatment candidate for DMD. Although earlier studies suggest a downregulation of PGC‐1α1 in dystrophic muscles,^[^ ^39^, ^45^, ^46^ ^]^ the expression levels of PGC‐1α4 in the DMD context remain unclear.

Intrigued by these findings, we sought to investigate the therapeutic effects of mRNA encoding PGC‐1α4 in mdx mice, a model for DMD. Initially, the dystrophic phenotypes in mdx mice were examined using a meticulously developed in vivo torque measurement technique, followed by the assessment of treatment outcomes after *Pgc‐1α4* mRNA administration using nanomicelles or LNPs. As shown later, we observed that nanomicelle‐delivered *Pgc‐1α4* mRNA significantly improved damage resistance and mitochondrial activities in dystrophic muscles. This study demonstrated the therapeutic potential of *Pgc‐1α4* mRNA for treating DMD while highlighting the importance of appropriate mRNA delivery systems.

## 2 Results

### 2.1 Impaired Damage Resistance upon Eccentric Contraction (ECC) in Dystrophic Mice

**Figure 1:**
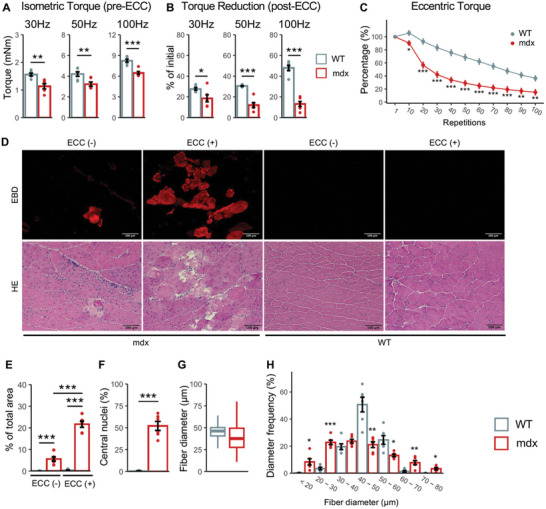
Functional and histological evaluation of dystrophic muscles in response to eccentric contraction (ECC). A) Mean isometric torque of the plantar flexors upon supramaximal stimulation (45 V and 0.5 ms) at incremental frequencies. B) Isometric torque measured post‐ECC, relative to the baseline torque per mouse from (A). C) Torque produced during 100 cycles of ECC, normalized to the torque in the first cycle. D) Representative images of Evans blue dye (EBD) staining (top panel) and hematoxylin and eosin (H&E) staining (bottom panel). E–H) Quantitative histological analyses. (E) Percentage of EBD‐positive area relative to total muscle section area. (F) Percentage of centrally nucleated fibers. (G) Fiber diameter boxplot. (H) Fiber diameter distribution across different sizes. For F‐H, ≈500 fibers per mouse were analyzed. WT and mdx refer to wild‐type mice and mdx mice. EBD, evans blue dye. Data are presented as mean ± SEM (*n* = 6 mice), and Welch's *t*‐test or one‐way ANOVA followed by Tukey's post hoc test were used (^***^ *p* < 0.001, ^**^ *p* < 0.01, and ^*^ *p* < 0.05).

Despite the widespread usage of mdx mice in DMD research, this animal model exhibits limitations, such as a milder muscle pathology and compensatory upregulation of genes like utrophin,^[^ ^47^ ^]^ which complicate the evaluation of therapeutic interventions. Several studies have reported a decline in damage resistance after repeated eccentric contractions in DMD model mice,^[^ ^48^, ^49^ ^]^ highlighting the utility of such protocols in assessing muscle fragility. Therefore, we adopted a rigorously established protocol to accurately analyze the strength of the plantar flexors (i.e., gastrocnemius, soleus, and plantaris), a crucial ambulatory muscle group, using in vivo torque measurement. This method measures the isometric torque following supramaximal electrical stimulation (45 V and 0.5 ms) before and after a damage protocol consisting of 100 ECC cycles, as described in Figure S1A,B (Supporting Information).^[^ ^18^, ^50^ ^]^ Given that supramaximal stimulation recruits all fibers in the muscle,^[^ ^51^, ^52^ ^]^ this approach allows the assessment of the true capacity of the muscle. We noted that the baseline isometric torque of mdx mice was slightly lower than that of wild‐type mice, although the difference was statistically significant (**Figure** **1**A). However, ECC sessions substantially diminished the isometric torque in mdx mice, resulting in a drastic torque reduction compared to wild‐type mice (Figure 1B; Figure S1C, Supporting Information). Specifically, under 100 Hz stimulation, the post‐ECC torque in mdx mice dropped to 13.18 ± 2.45% of the initial pre‐ECC levels, in contrast to 47.93 ± 2.53% in their wild‐type counterparts (*p* < 0.0001). Moreover, the eccentric torque in mdx mice was extensively lower throughout the ECC fatigue protocol (Figure 1C; Figure S1D, Supporting Information). Consistent with previous findings, these findings reflect a lower muscle strength and damage resistance level in mdx muscles.

We also examined muscle histology using Evans blue dye (EBD), a membrane permeability marker, whose presence in muscle fibers represents sarcolemma damage.^[^ ^53^ ^]^ EBD staining presented distinct outcomes in mdx mice subjected to ECC damage protocol versus those without ECC. In ECC‐free mdx mice, signals of EBD uptake were only occasionally observed. Nonetheless, ECC exposure resulted in a remarkable increase in EBD accumulation (from 5.56 ± 0.97% to 21.67 ± 1.45% of the total fiber area, *p* < 0.0001), indicating exacerbated sarcolemma damage and muscle fiber injury (Figure 1D,E). On the other hand, in wild‐type mice, ECC‐induced EBD uptake was minimal and showed no significant difference from the non‐ECC group. The EBD signals corresponded to the fibers undergoing necrosis or degeneration in H&E staining (Figure 1D, bottom panel). Analysis of H&E staining images in the non‐ECC group implied a higher proportion of centrally nucleated fibers, a smaller average fiber size, and a higher degree of heterogeneity in fiber size (Figure 1F–H), likely due to the enhanced muscle regeneration in mdx mice.^[^ ^47^ ^]^ These data reveal the susceptibility to ECC‐induced stress and the inherent fiber fragility of dystrophin‐deficient muscles.

### 2.2 Lower Levels of PGC‐1α Isoforms and Associated Genes were Observed in Dystrophic Muscles

**Figure 2:**
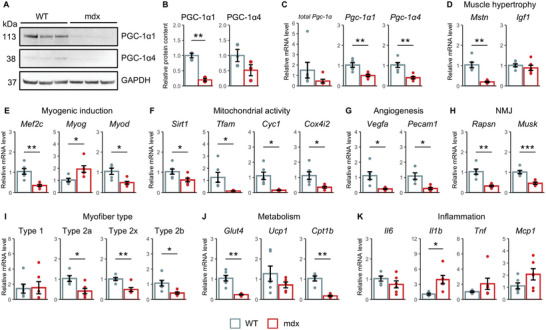
Expression levels of PGC‐1α and associated genes in dystrophic muscles. A) Western blotting of PGC‐1α1 and PGC‐1α4 protein levels (*n* = 3 mice). B) Quantitative analysis of the western blot bands normalized to GAPDH. C) Relative mRNA expression levels of total Pgc‐1α (Pgc‐1α1,2,3, and 4), Pgc‐1α1, and Pgc‐1α4. D–K) Relative mRNA levels of genes associated with PGC‐1α in various aspects including muscle hypertrophy (D), myogenic induction (E), mitochondrial activity (F), angiogenesis (G), neuromuscular junction (NMJ) (H), myofiber type (I), metabolism (J), and inflammation (K). All analyses were performed using plantar flexor muscles. Data are presented as mean ± SEM (*n* = 6 mice), and Welch's *t*‐test was used (^***^ *p* < 0.001, ^**^ *p* < 0.01, and ^*^ *p* < 0.05).

As previous findings highlight PGC‐1α as the key protein underpinning skeletal muscle damage resistance^[^ ^18^, ^19^ ^]^ we investigated the expression levels of its isoforms in mdx muscles. In agreement with previous studies,^[^ ^39^, ^45^ ^]^ we found a downregulation at both mRNA and protein levels in dystrophic mice (**Figure** **2**A–C). Moreover, our further investigation extended to the putative PGC‐1α interacting genes (illustrated in Figure S2, Supporting Information). These genes, which encompass a range of proteins involved in various aspects, such as muscle regeneration,^[^ ^22^, ^54^ ^]^ energy metabolism,^[^ ^19^, ^20^, ^24^, ^28^ ^]^ mitochondrial function,^[^ ^32^, ^33^, ^55^ ^]^ myofiber type switching,^[^ ^36^, ^56^ ^]^ neuromuscular junction,^[^ ^31^, ^34^, ^57^ ^]^ and inflammation^[^ ^58^, ^59^, ^60^ ^]^ also exhibited notably dysregulated levels in mdx mice (Figure 2D–K). These observations collectively demonstrate a compromised expression level of PGC‐1α1, PGC‐1α4, and their associated genes in dystrophic mice.

### 2.3 Preparation of mRNAs Encoding PGC‐1α4

Prior studies involving the overexpression of PGC‐1α1 in DMD models have indicated beneficial effects,^[^ ^34^, ^35^, ^36^, ^37^, ^38^, ^39^, ^40^ ^]^ but the potential therapeutic advantages of PGC‐1α4 in DMD are yet to be determined. Recently, Guo et al., demonstrated that AAV‐mediated PGC‐1α4 delivery in muscle could alleviate aging‐related muscular dystrophy.^[^ ^44^ ^]^ Based on these findings, we hypothesized that restoration of PGC‐1α4 via mRNA could offer therapeutic benefits to mdx mice. An earlier protocol was utilized to synthesize mRNA in vitro, as depicted in Figure S3A (Supporting Information).^[^ ^61^ ^]^ The length of the template DNA for *Pgc‐1α4* mRNA was 1098 bp, as verified by gel electrophoresis (Figure S3B, Supporting Information). Additionally, automated mRNA electrophoresis validated the size, integrity, and purity of the synthesized mRNA (Figure S3C, Supporting Information). Expression of PGC‐1α4 was detected at 38 kDa in HEK293 cells 24 h after mRNA transfection (Figure S3D, Supporting Information). These experiments confirmed the authenticity of the synthesized *Pgc‐1α4* mRNA.

### 2.4 Evaluation of Luciferase Activity Following *Fluc* mRNA Delivery Mediated by Polyplex Nanomicelle

**Figure 3:**
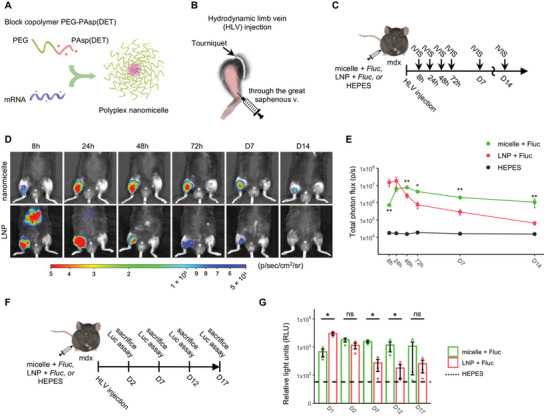
Characterization of protein expression after hydrodynamic limb vein (HLV) injection of mRNA‐loaded nanomicelles or LNPs. A) Schematic illustration of the formation of polyplex nanomicelles resulting from the interaction of mRNA and the block copolymer PEG‐PAsp(DET). B) Graphic representation of the HLV administration technique. C–G) Evaluation of luciferase expression from Fluc mRNA‐loaded nanomicelles or LNPs following HLV administration in mdx mice. (C) Experimental design of (D,E). (D) Representative bioluminescence images. Color bar represents the radiance intensity. (E) Time course of luciferase expression was observed at multiple time points by IVIS. (F) Experimental design of (G). (G) Luciferase activity was measured at different time points by luciferase assay. IVIS, in vivo imaging system. HEPES was used as a control injection in the contralateral limb. Data are presented as mean ± SEM (*n* = 3 mice), and log transformation was performed in (F) and (G). One‐way ANOVA followed by Tukey's post hoc test was used (^**^ *p* < 0.01 and ^*^ *p* < 0.05, micelle + Fluc vs. LNP + Fluc; ns indicates non‐significant).

For mRNA delivery into the muscle, we performed hydrodynamic limb vein (HLV) injection, which involved temporary isolation of the limb and injection of a large volume of mRNA solution through the great saphenous vein (**Figure** **3**B). This injection method has been reported to promote homogeneous and widespread mRNA uptake in the muscle fiber with minimal tissue damage^[^ ^16^, ^62^, ^63^, ^64^ ^]^ and was well tolerated in patients with muscular dystrophy.^[^ ^65^, ^66^ ^]^

To assess the protein production from mRNA delivered by nanomicelles or LNPs via HLV injection, we employed *Fluc* mRNA, in vivo imaging system, and luciferase assay (IVIS; Figure 3C,F). In line with our prior observations in wild‐type mice,^[^ ^16^ ^]^ *Fluc* mRNA‐loaded nanomicelles resulted in prolonged luciferase expression, which peaked at day 2 and lasted up to two weeks in mdx mice (Figure 3D,E). In contrast, LNPs showed a strong luciferase expression within the first 24 h, together with significant off‐target expression in the liver. However, the expression from LNPs rapidly decreased at 48 h post‐administration. The luciferase assay results were consistent with the trends observed in the IVIS data, despite differences in statistical significance due to the varying sensitivities of the two methods (Figure 3F,G). These results demonstrate that while LNPs enabled a significantly higher initial expression, nanomicelles provided a longer duration of protein expression and more localized delivery.

### 2.5 Improvement of Damage Resistance in Dystrophic Mice by Nanomicelle‐Delivered *Pgc‐1α4* mRNA

**Figure 4:**
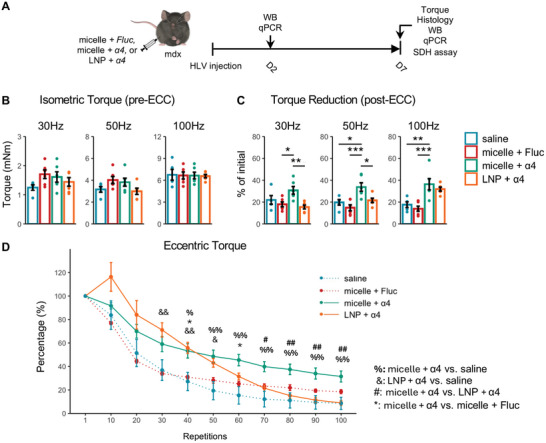
Improvement of muscle damage resistance in dystrophic mice following nanomicelle‐delivevred Pgc‐1α4 mRNA treatment. A) Schematic illustration of the experiment design. B–D), Assessment of plantar flexor muscle performance after injection. (B) Mean isometric torque of the plantar flexors upon supramaximal stimulation at incremental frequencies (*n* = 5 for the saline group; *n* = 6 for all other groups). (C) Isometric torque measured post‐ECC relative to the baseline torque per mouse from (B). (D) Torque produced during 100 cycles of ECC normalized to the torque in the first cycle (*n* = 5 for the saline and micelle + Fluc groups; *n* = 6 for all other groups). Data are presented as mean ± SEM, and one‐way ANOVA followed by Tukey's post hoc test was used (^***^ *p* < 0.001, ^**^ *p* < 0.01, and ^*^ *p* < 0.05).

Next, we sought to investigate whether *Pgc‐1α4* mRNA could alleviate the muscle fragility in mdx mice. To this end, four groups were established: 1) saline, 2) nanomicelle‐delivered *Fluc* mRNA (micelle + *Fluc*), 3) nanomicelle‐delivered *Pgc‐1α4* mRNA (micelle + *α4*), and 4) LNP‐delivered *Pgc‐1α4* mRNA (LNP + *α4*). These mRNA formulations or the saline control were injected via the HLV approach, and treatment outcomes were evaluated through muscle torque, histology, gene expression, and SDH assay (**Figure** **4**A).

One week after the administration, we observed that the three groups exhibited similar levels of pre‐ECC isometric torque (Figure 4B). Nonetheless, while nanomicelle‐delivered *Pgc‐1α4* mRNA resulted in notably increased post‐ECC torque compared to the control group, LNP‐delivered *Pgc‐1α4* mRNA did not yield similar improvements (Figure 4C; Figure S6A, Supporting Information). For instance, under 30 Hz stimulation, the post‐ECC torque was reduced to 30.71 ± 3.47% of the initial value in the nanomicelle‐delivered *Pgc‐1α4* mRNA group, compared to 21.99 ± 3.96% in mice receiving saline (not significant), 17.94 ± 2.27% in mice receiving nanomicelle‐delivered *Fluc* mRNA (*p* = 0.0236) and 15.48 ± 1.78% in mice receiving LNP‐delivered *Pgc‐1α4* mRNA (*p* = 0.0062). When the stimulation was increased to 100 Hz, the nanomicelle‐delivered *Pgc‐1α4* mRNA group saw a reduction to 36.33 ± 5.15%, compared to 17.62 ± 2.61% in the saline control group (*p* = 0.0049), 13.91 ± 2.35% in the nanomicelle‐delivered *Fluc* mRNA group (*p* = 0.0005) and 31.85 ± 1.85% in the LNP‐delivered *Pgc‐1α4* mRNA group (not significant). Additionally, the highest level of eccentric torque during the latter part of the damage protocol was found in the nanomicelle‐delivered *Pgc‐1α4* mRNA group, indicating enhanced damage resistance than the other two groups (Figure 4D; Figure S6B, Supporting Information). These data suggest that while nanomicelle‐delivered *Pgc‐1α4* mRNA improved damage resistance in mdx muscles, LNP‐mediated delivery did not provide the same level of therapeutic benefit.

### 2.6 Reduction of ECC‐Induced Myofiber Damage Following Nanomicelle‐Delivered *Pgc‐1α4* mRNA Treatment

**Figure 5:**
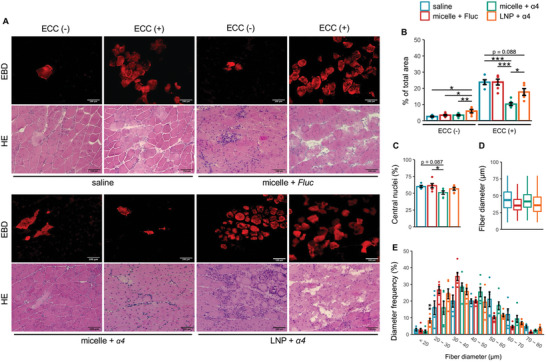
Reduction of myofiber damage in dystrophic mice following nanomicelle‐delivevred Pgc‐1α4 mRNA treatment. A) Representative images of EBD staining (upper panel) and H&E staining (lower panel) post‐treatment. B–E), Quantitative histological analyses. (B) Percentage of EBD‐positive area. (C) Percentage of centrally nucleated fibers. (D) Fiber diameter boxplot. (E) Fiber diameter distribution across different sizes. Data are presented as mean ± SEM (*n* = 5 for the saline and LNP + α4 groups; *n* = 6 for all other groups), and one‐way ANOVA followed by Tukey's post hoc test was used (^***^ *p* < 0.001, ^**^ *p* < 0.01, and ^*^ *p* < 0.05). For (B–E), 300–500 fibers per mouse were analyzed.

Following torque measurements, we conducted histological examinations to assess myofiber damage. The results revealed that while there was no difference in EBD uptake between the groups receiving nanomicelle‐delivered *Fluc* or *Pgc‐1α4* mRNA, LNP‐delivered *Pgc‐1α4* mRNA administration led to a slightly increased level of EBD signals even in the absence of ECC damage protocol, implying an elevated level of baseline fiber damage (**Figure** **5**A,B). This is likely related to LNP‐induced inflammation, as evidenced by the accumulation of immune cells observed in the H&E staining (Figure 5A, bottom panel). For the groups exposed to ECC damage, mice receiving nanomicelle‐delivered *Pgc‐1α4* mRNA exhibited the lowest level of EBD uptake (10.26 ± 0.93%) compared to that of the ones receiving saline (23.80 ± 1.48%, *p* < 0.0001), nanomicelle‐delivered *Fluc* mRNA (23.85 ± 1.82%, *p* < 0.0001) and LNP‐delivered *Pgc‐1α4* mRNA (17.71 ± 2.13%, *p* = 0.0215). This is also corroborated by a reduction in the areas of degenerating fibers observed in H&E staining (Figure 5A, bottom panel). Histological analysis of the H&E staining images in the ECC‐free groups suggested that nanomicelle‐delivered *Pgc‐1α4* mRNA treatment was associated with fewer central nuclei (Figure 5C). These findings indicate that nanomicelle‐delivered *Pgc‐1α4* mRNA confers a protective effect in attenuating ECC‐induced myofiber injury.

### 2.7 Enhanced Gene Expression and Mitochondrial Activity After Nanomicelle‐Delivered *Pgc‐1α4* mRNA Administration

**Figure 6:**
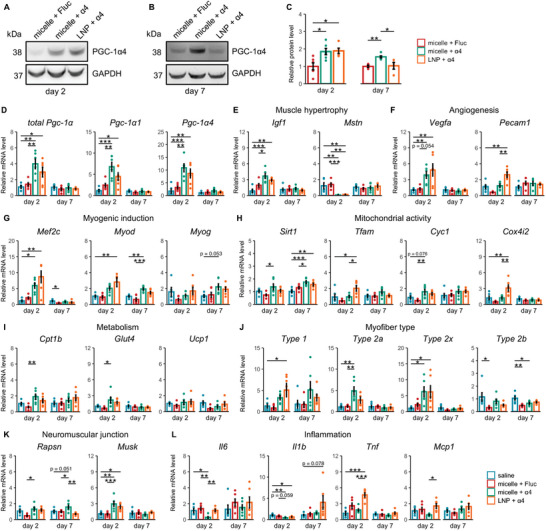
General upregulation of dysregulated genes after nanomicelle‐delivered Pgc‐1α4 mRNA treatment. A,B) Western blotting images of PGC‐1α4 protein expression on day 2 (A) and day 7 (B). C) Quantification of western blotting bands normalized to GAPDH (for day 2, *n* = 5 for the LNP + α4 group, *n* = 6 for all other groups; for day 7, *n* = 4 for the LNP + α4 group, *n* = 5 for the micelle + Fluc group, and *n* = 6 for the micelle + α4 group). D–L) Relative mRNA levels of genes associated with PGC‐1α on day 2 and day 7. All analyses were performed using plantar flexor muscle samples. Data are presented as mean ± SEM (*n* = 6 mice), and one‐way ANOVA followed by Tukey's post hoc test was used (^***^ *p* < 0.001, ^**^ *p* < 0.01, and ^*^ *p* < 0.05).

We then analyzed the expression levels of PGC‐1α and its associated genes on both day 2 and day 7 after a single injection of 10 µg mRNA. We selected Day 2 and Day 7 to capture key differences in expression profiles based on our observations in Figure 3D,E. Day 2 marks the transient LNP expression peak, while Day 7 provides a midpoint where LNP expression diminished and nanomicelle expression remained stable. Western blot analysis revealed detectable PGC‐1α4 protein levels from nanomicelle at both time points (*p* = 0.0233 for day 2; *p* = 0.0052 for day 7), whereas LNP showed detectable expression only on day 2 (*p* = 0.0241), with day 7 levels nearly equivalent to the control group (**Figure** **6**A–C). On day 2, a general upregulation of genes dysregulated in dystrophic mice was observed, signifying a robust and immediate response to nanomicelle‐mediated *Pgc‐1α4* mRNA delivery (Figure 6). In particular, an elevation was found in the expression levels of *Igf1*, *Glut4*, and *Vegfa*, previously identified PGC‐1α4 targets.^[^ ^41^, ^42^, ^43^ ^]^ Interestingly, there was an almost five‐fold increase in the transcript level of PGC‐1α1 after nanomielle‐delivered *Pgc‐1α4* mRNA administration (*p* = 0.0002, micelle + α4 vs saline; Figure 6D). Meanwhile, LNP‐delivered *Pgc‐1α4* mRNA had similar or even higher upregulating effects on a few genes such as *Mef2c*, *Cox4i2*, and *Pecam1*, which is likely attributed to its strong initial expression. Surprisingly, we observed that nanomicelle‐delivered *Pgc‐1α4* mRNA significantly mitigated the expression of several inflammatory markers such as *Il6* (*p* = 0.0171, micelle + α4 vs saline; *p* = 0.0012, micelle + α4 vs micelle + Fluc), whereas LNP‐delivered *Pgc‐1α4* mRNA did not have such effects (Figure 6L). It has been reported that PGC‐1α and its variants could alleviate inflammation and prevent cell apoptosis,^[^ ^58^, ^59^, ^67^ ^]^ presumably by repressing the nuclear factor κB (NFκB).^[^ ^60^, ^68^ ^]^ This implies that the anti‐inflammatory effects of *Pgc‐1α4* mRNA were abolished when delivered by LNPs, potentially due to the inflammation induced by the LNPs themselves.

By day 7, the upregulating effect by both nanomicelle‐delivered and LNP‐delivered *Pgc‐1α4* mRNA was narrowed down to only a few genes. However, one of the exceptions was the significant upregulation of *Sirt1* (Figure 6H, *p* = 0.0272, micelle + α4 vs micelle + Fluc; *p* = 0.0003, micelle + α4 vs saline; *p* = 0.0060, LNP + α4 vs saline), a pivotal gene involved in muscle metabolism.^[^ ^69^ ^]^ While SIRT1 is known to activate PGC‐1α1,^[^ ^70^ ^]^ whether it interacts with the shorter isoform PGC‐1α4 remains to be elucidated. Our findings imply that PGC‐1α4 may improve muscle damage resistance by supporting mitochondrial function and energy metabolism.

**Figure 7:**
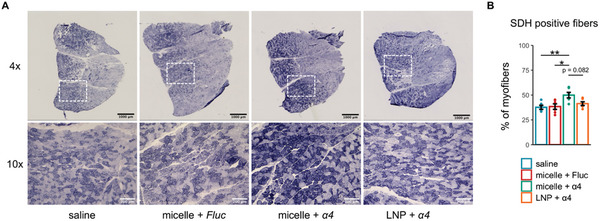
Enhancement of mitochondrial activity by nanomicelle‐deliverd Pgc‐1α4 mRNA treatment. A) Representative succinate dehydrogenase (SDH) assay images. B) Quantitative analysis of the percentage of SDH‐positive fibers. Data are presented as mean ± SEM (*n* = 5 for the saline group; *n* = 6 for all other groups), and one‐way ANOVA followed by Tukey's post hoc test was used (^*^ *p* < 0.05 and ^**^ *p* < 0.01).

To further investigate the impact of *Pgc‐1α4* mRNA on mitochondrial activity, we performed the succinate dehydrogenase (SDH) assay, which indicates mitochondrial oxidative capacity.^[^ ^71^ ^]^ The results showed that mice treated with nanomicelle‐delivered *Pgc‐1α4* mRNA exhibited a pronounced increase in SDH staining intensity across muscle sections (**Figure** **7**A). Quantitative analysis revealed that there was an ≈10% rise in SDH‐positive fibers in the nanomicelle‐delivered *Pgc‐1α4* mRNA group compared to the saline control (49.90 ± 2.75% vs 37.64 ± 2.02%, *p* = 0.0078) or the nanomicelle‐delivered *Fluc* mRNA group (49.90 ± 2.75% vs 38.38 ± 2.72%, *p* = 0.0130, Figure 7B). Moreover, there was a trend toward a higher percentage of SDH‐positive fibers in the nanomicelle‐delivered *Pgc‐1α4* mRNA group versus the LNP‐delivered *Pgc‐1α4* mRNA group, although this difference was not statistically significant (*p* = 0.082). Taken together, these results further support the effectiveness of nanomicelle‐delivered *Pgc‐1α4* mRNA in enhancing mitochondrial activity in dystrophic muscles.

## 3 Discussion

In this study, we investigated the potential of mRNA therapy encoding PGC‐1α4 to treat dystrophic mdx mice. Our approach encompassed in vivo torque measurement, histological analysis, and gene expression profiling. Additionally, SDH staining was performed to examine mitochondrial activity. The results revealed notable improvements in damage resistance and mitochondrial activity in dystrophic muscles treated with nanomicelle‐delivered *Pgc‐1α4* mRNA, marking an initial proof‐of‐concept for the viability of mRNA‐based therapies in addressing neuromuscular diseases. Furthermore, we would like to propose the concept of a combined treatment regimen. When combined with existing methods such as exon‐skipping and AAV‐mediated micro‐dystrophin gene transfer, mRNA therapy could potentially overcome the drawbacks of these modalities and help achieve better therapeutic outcomes. While the investigation of this proposal was beyond the scope of this study, our findings provide a solid foundation for subsequent research on this topic.

In evaluating the effects of nanomicelle‐delivered *Pgc‐1α4* mRNA, our findings suggest that while there was a general increase in dysregulated genes involved in DMD, the observed upregulation was diminished by day 7 (Figure 6), indicating the transient nature of such therapeutic benefits. It is imperative to acknowledge that, although our current study was confined to evaluations up to day 7, it is crucial to consider the mid‐to‐long‐term therapeutic implications. This is particularly relevant when juxtaposed with LNP, the most clinically advanced mRNA delivery platform. In line with our earlier findings,^[^ ^16^ ^]^ this study revealed that mRNA delivered via LNP exhibited an even shorter expression window when compared to that delivered by polyplex nanomicelles under the same settings (Figure 3D–G). Future explorations could likely benefit from using novel mRNA structures such as circular RNA (circRNA) or self‐amplifying RNA (saRNA) to prolong the duration of protein expression.^[^ ^72^, ^73^, ^74^, ^75^ ^]^ The prospect of sustained protein expression (a month, for instance), coupled with repeated dosing, could transform mRNA therapy into a practical treatment for muscular dystrophies and other neuromuscular diseases.

PGC‐1α4, a shorter variant of PGC‐1α1 whose functions are still under investigation, is thought to promote muscle hypertrophy, anaerobic glycolysis, and angiogenesis.^[^ ^41^, ^42^ ^]^ In this study, an elevated level of SIRT expression was observed for up to one week following nanomicelle‐delivered *Pgc‐1α4* mRNA treatment, indicating that PGC‐1α4 may exert its protective effects through the upregulation of *Sirt1* (Figure 6H). By activating key metabolic regulators such as PGC‐1α1, SIRT1 can increase mitochondrial biogenesis and improve the muscle's ability to generate ATP efficiently.^[^ ^28^, ^70^ ^]^ Specifically, in the circumstance of DMD, muscle‐specific overexpression of SIRT1 in mdx mice led to an increase in PGC‐1α1 content and ameliorated dystrophic phenotypes.^[^ ^76^ ^]^ Interestingly, we also observed an upregulated transcript level of PGC‐1α1 two days after nanomicelle‐delivered *Pgc‐1α4* mRNA administration (Figure 6D), although it remains uncertain whether this increase is directly mediated by PGC‐1α4 or through the involvement of SIRT1. This suggests that a synergistic relationship among PGC‐1α4, SIRT1, and PGC‐1α1 may be involved in the molecular mechanism driving the observed treatment outcomes. Achieving such effects with the shorter *Pgc‐1α4* mRNA (≈1100 nt) is advantageous from the perspective of mRNA synthesis on an industrial level.

Another factor underlying the excellent therapeutic effect is attributed to the injection method of mRNA, the HLV injection. This injection method facilitates the introduction of mRNAs to the target muscles in a diffusive manner,^[^ ^62^, ^63^ ^]^ where PGC‐1α4 is expected to be expressed in most muscle fibers in the lower limb with minimal tissue damage.^[^ ^64^, ^77^ ^]^ However, it is essential to recognize that DMD is a systemic disease, and local treatments may not fully address the extensive symptoms such as those in the diaphragm and cardiac muscles. This highlights the need for more sophisticated delivery methods to introduce mRNA into various target muscles. On the other hand, the positive outcomes observed with mRNA therapy in local treatment hold promise for its application in a spectrum of other muscular diseases or injuries.

## 4 Conclusion

In conclusion, this study demonstrates that nanomicelle‐delivered *Pgc‐1α4* mRNA enhanced muscle damage resistance and mitochondrial activity in a DMD mouse model. As the first proof‐of‐concept, these findings showcase its capability to overcome the limitations of existing therapies and represent a foundational step toward developing mRNA‐based therapeutics for neuromuscular diseases.

## 5 Experimental Section

### Animals

C57BL/6 mice were sourced from Nihon Crea (Tokyo, Japan). Mdx mice with C57BL/10 background, backcrossed ten times into C57BL/6 genetic background to ensure intact Toll‐like receptor 4 (TLR4) and reliable modeling of immune responses,^[^ ^78^ ^]^ were utilized for this study. Animals were acclimated for at least 7 days in the National Center of Neurology and Psychiatry's (NCNP) animal facility under standard conditions. All experiments were initiated with male mice aged 10 weeks, with each experiment lasting 1 to 2 weeks. All animal procedures received approval from NCNP's Experimental Animal Care and Use Committee (Approval ID: 2022008R8).

### Muscle Torque Measurement

In vivo muscle torque measurements were conducted following an established protocol (described in Figure S1A,B, Supporting Information) to assess the plantar flexor muscles’ functional capacity (i.e., gastrocnemius, soleus, and plantaris).^[^ ^18^, ^50^, ^79^, ^80^ ^]^ Anesthetized with 2% isoflurane, mice were positioned on a platform to align one hind paw at a 90° angle to the tibia on a sensor footplate (model S‐14154, Takei Scientific Instruments, Niigata, Japan). Supramaximal stimulation (45 V and 0.5 ms) at frequencies of 30, 50, and 100 Hz was delivered to the plantar flexor muscles through a pair of surface electrodes. The in vivo damage protocol consisted of 100 cycles of eccentric contractions (ECC; 40° dorsiflexion at 150°/s) combined with supramaximal electrical stimulation (45 V, 0.5 ms, and 50 Hz). The unstimulated contralateral plantar flexors served as controls. Torque data were recorded and analyzed with LabChart (v8.1.22, ADInstruments, Dunedin, New Zealand).

### Synthesis of mRNA

An overview of mRNA synthesis process is depicted in Figure S3A (Supporting Information) following a previous protocol.^[^ ^61^ ^]^ The sequence of *Pgc‐1α4* mRNA was obtained from the NCBI database (accession number JX866948). Codon‐optimized oligonucleotides, including 5′ and 3′ untranslated regions (UTRs, detailed in Figure S4, Supporting Information), were inserted into a pMA‐RQ (AmpR) backbone (Thermo Fisher Scientific, Waltham, MA, USA). Linear DNA templates, which contain a T7 RNA polymerase promoter and a poly(A) tail, were then prepared from the plasmid by polymerase chain reaction (PCR) using PrimeSTAR Max DNA Polymerase (Takara Bio Inc., Shiga, Japan) and UTR‐specific primers. Synthesis of mRNA was achieved through in vitro transcription (IVT), which included a capping step to boost the translation efficiency.^[^ ^7^, ^9^ ^]^ Additionally, complete substitution with N1‐methylpseudourine (m1Ψ) was employed to reduce the innate immunogenicity of mRNA.^[^ ^81^, ^82^, ^83^ ^]^ IVT was performed using the MEGAscript T7 Transcription Kit (Thermo Fisher Scientific), CleanCap Reagent AG (N‐7113, TriLink BioTechnologies, San Diego, CA, USA), and N1‐methylpseudouridine‐5′‐triphosphate (N‐1081, TriLink BioTechnologies). After the completion of IVT, the synthesized mRNA was dephosphorylated using Quick CIP (New England Biolabs, Ipswich, MA, USA) and purified using an RNeasy Mini Kit (Qiagen, Hilden, Germany). The quality of mRNA was confirmed on an Agilent 2100 Bioanalyzer (Agilent Technologies, Santa Clara, CA, USA). *Fluc* mRNA (with 5moU modification) was purchased from TriLink BioTechnologies.

### mRNA Transfection

HEK293 cells were cultured in DMEM supplemented with 10% fetal bovine serum (FBS) and 1% penicillin‐streptomycin in a humidified incubator at 37 °C with 5% CO_2_. Cells were transfected with mRNA at 70–90% confluency using Lipofectamine MessengerMAX (Thermo Fisher Scientific) according to the manufacturer's protocol. Cells were harvested 24 h post‐transfection for subsequent analysis.

### Preparation of mRNA‐Loaded Polyplex Nanomicelles and LNPs

The block copolymer composed of polyethylene glycol (PEG) and polyamino acid (Poly{N‐[N′‐(2‐aminoethyl)‐2‐aminoethyl]aspartamide}) (PEG‐PAsp(DET)) was synthesized in accordance with an established protocol.^[^ ^15^ ^]^ The molecular weight of PEG was set at 43 000 g mol^−1^, and the PAsp(DET) polymerization degree was 63, as confirmed by 1H NMR analysis. For mRNA‐loaded polyplex nanomicelle formation, the block copolymer PEG‐PAsp(DET) and mRNA were each dissolved in 10 mm HEPES and then mixed at an N:P ratio, that is, the ratio of positively charged amines in the polymer to negatively charged phosphates in the mRNA, of 8:1 (Figure 4A). The mRNA concentration was adjusted to 33.33 µg mL^−1^ in the final solution. LNPs composed of SM‐102, DMG‐PEG(2000), 1,2‐DSPC, and cholesterol (Figure S5, Supporting Information) were formulated using the LipidLaunch LNP‐102 Exploration Kit (Cayman Chemical, MI, USA). The LNP solution was concentrated by ultrafiltration using 100 kDa Amicon Ultra‐15 Centrifugal Filters (UFC910008, Merck Millipore, MA, USA). The physicochemical properties of mRNA‐loaded polyplex nanomicelles and LNPs are listed in Table S1 (Supporting Information).

### In Vivo mRNA Administration

Hydrodynamic limb vein injection was performed as previously described^[^ ^62^ ^]^ and illustrated in Figure 4B. Briefly, mice were anesthetized with 2% isoflurane, and a tourniquet was applied proximally on the hindlimb. A 300 µL solution containing 10 µg mRNA was injected into the great saphenous vein using a 30G needle. The tourniquet was maintained for 5 min to facilitate distribution of the mRNA solution. A thermal pad was used to maintain the body temperature of the mice.

### In Vivo Imaging and Luciferase Assay

Luciferase expression was evaluated using the IVIS Lumina II system (Xenogen, Almeda, CA, USA) at multiple time points following *Fluc* mRNA injection. Mice were anesthetized with 2% isoflurane and then received an intraperitoneal injection of 3 mg D‐luciferin (Wako, Osaka, Japan). Images were acquired 10 min after D‐luciferin injection with an exposure time of 60 s. At the end of the in vivo imaging experiments, mice were euthanized for muscle collection, and luciferase activity was quantified using the Luciferase Assay System (Promega, Madison, WI, USA). Briefly, the collected muscles were snap‐frozen in liquid nitrogen and homogenized with Passive Lysis Buffer (Promega) using a Multibeads Shocker (Yasuikikai, Osaka, Japan). The homogenate was centrifuged at 12 000 rpm for 5 min at 4 °C, and the supernatant was used to measure luciferase activity with a GloMax Navigator Microplate Luminometer (Promega).

### Western Blotting

Cell and tissue lysates were prepared by homogenization in RIPA buffer containing protease and phosphatase inhibitors. Protein concentrations were determined using the Micro BCA Protein Assay Kit (Thermo Fisher Scientific) before loading onto a Bolt 4–12% Bis‐Tris Plus gel (Thermo Fisher Scientific) for electrophoresis and subsequent transfer to PVDF membranes. Membranes were blocked with 5% non‐fat dry milk and incubated with primary antibodies against PGC‐1α (sc‐518025, Santa Cruz; ST1202, Calbiochem) and loading controls GAPDH (D16H11, Cell Signaling Technology) and α‐tubulin (T6199, Sigma–Aldrich). After incubation with an HRP‐conjugated secondary antibody (W402B, Promega), bands were visualized by chemiluminescence. Membranes were washed three times with TBST between incubations.

### Quantitative Real‐Time Polymerase Chain Reaction (qRT‐PCR)

All analyses were performed using plantar flexor muscles (i.e., gastrocnemius and soleus). Total RNA was isolated using the RNeasy Fibrous Tissue Mini Kit (Qiagen). After reverse transcription (ReverTra Ace qPCR RT Kit, TOYOBO, Osaka, Japan), cDNA was mixed with PowerTrack SYBR Green Master Mix (Applied Biosystems, Waltham, CA, USA) and the gene‐specific primers listed in Table S2 (Supporting Information). Ct values were obtained on a StepOnePlus real‐time PCR system (Applied Biosystems), and relative mRNA levels were calculated by normalizing to the housekeeping gene *Actb* using the ∆∆ Ct method.

### Histology and Image Analysis

Muscle tissues were flash‐frozen in pre‐cooled isopentane and sectioned at 6 µm thickness. For Evans Blue staining, mice received an intraperitoneal injection of 10% Evans blue dye (EBD) in PBS 24 h prior to sampling. EBD permeation was visualized by its red autofluorescence using a fluorescence microscope (BZ‐X700, Keyence, Osaka, Japan). For succinate dehydrogenase (SDH) staining, fresh cryosections were incubated in a solution containing nitro blue tetrazolium chloride (N5514, Sigma–Aldrich) and sodium succinate (S2378, Sigma–Aldrich), then dehydrated and mounted. Histological images were analyzed in ImageJ (version 1.53k).

### Statistical Analysis

All data were expressed as mean ± standard error of the mean (SEM). Statistical significance was assessed by one‐way ANOVA followed by Tukey's *post hoc* test or Welch's *t*‐test (^***^ *p* < 0.001, ^**^ *p* < 0.01, and ^*^ *p* < 0.05). Data were analyzed and visualized with Rstudio (version 2023.06.0+421).

## Conflict of Interest

The authors declare no conflict of interest.

## Author Contributions

X.D.: performed conceptualization, data curation, formal analysis, investigation, visualization, writing—original draft, wrote—reviewed and edited the final manuscript. H.N. and Y.S.: performed methodology. T.Y.: performed conceptualization, methodology, wrote—reviewed, and edited the final manuscript. K.M. and N.M.: project administration. Y.A.: conceptualization, project administration, acquired funding acquisition, wrote—reviewed, and edited the final manuscript. K.I.: project administration, acquired funding acquisition, wrote—reviewed, and edited the final manuscript.

## Supporting information



Supporting Information

## Data Availability

The data that support the findings of this study are available from the corresponding author upon reasonable request.
