# Supplementary material for: Polyplex Nanomicelle‐Mediated Pgc‐1α4 mRNA Delivery Via Hydrodynamic Limb Vein Injection Enhances Damage Resistance in Duchenne Muscular Dystrophy Mice
Source: Adv Sci (Weinh). 2025 Mar 6;12(16):2409065. doi: 10.1002/advs.202409065 (PMC12021044; doi:10.1002/advs.202409065)
Supplement: Supplementary file 1 — Supporting Information [file ADVS-12-2409065-s001.pdf]

ADVANCED SCIENCE
Open Access

Supporting Information

for *Adv. Sci.*, DOI 10.1002/advs.202409065

Polyplex Nanomicelle-Mediated *Pgc-1α4* mRNA Delivery Via Hydrodynamic Limb Vein Injection Enhances Damage Resistance in Duchenne Muscular Dystrophy Mice

*Xuan Du, Hideyuki Nakanishi, Takashi Yamada, Yooksil Sin, Katsura Minegishi, Norio Motohashi, Yoshitsugu Aoki and Keiji Itaka**

Polyplex nanomicelle-mediated *Pgc-1α4* mRNA delivery via hydrodynamic limb vein injection enhances damage resistance in Duchenne muscular dystrophy mice

*Xuan Du[1], Hideyuki Nakanishi[1, 4], Takashi Yamada[2], Yooksil Sin[1, 4], Katsura Minegishi[3], Norio Motohashi[3], Yoshitsugu Aoki[3], and Keiji Itaka[1, 4, *]*

[1] Department of Biofunction Research, Laboratory for Biomaterials and Bioengineering, Institute of Science Tokyo, Tokyo 101-0062, Japan

[2] Department of Physical Therapy, Sapporo Medical University, Sapporo 060-8556, Japan

[3] Department of Molecular Therapy, National Institute of Neuroscience, National Center of Neurology and Psychiatry (NCNP), Tokyo 187-8502, Japan

[4] Clinical Biotechnology Team, Center for Infectious Disease Education and Research (CiDER), Osaka University, Osaka 565-0871, Japan

* Correspondence:

Prof. Keiji Itaka

Department of Biofunction Research,

Laboratory for Biomaterials and Bioengineering,

Institute of Science Tokyo

2-3-10 Kanda-Surugadai, Chiyoda, Tokyo 101-0062, Japan

Tel.: +81-3-5280-8087

itaka.bif@tmd.ac.jp

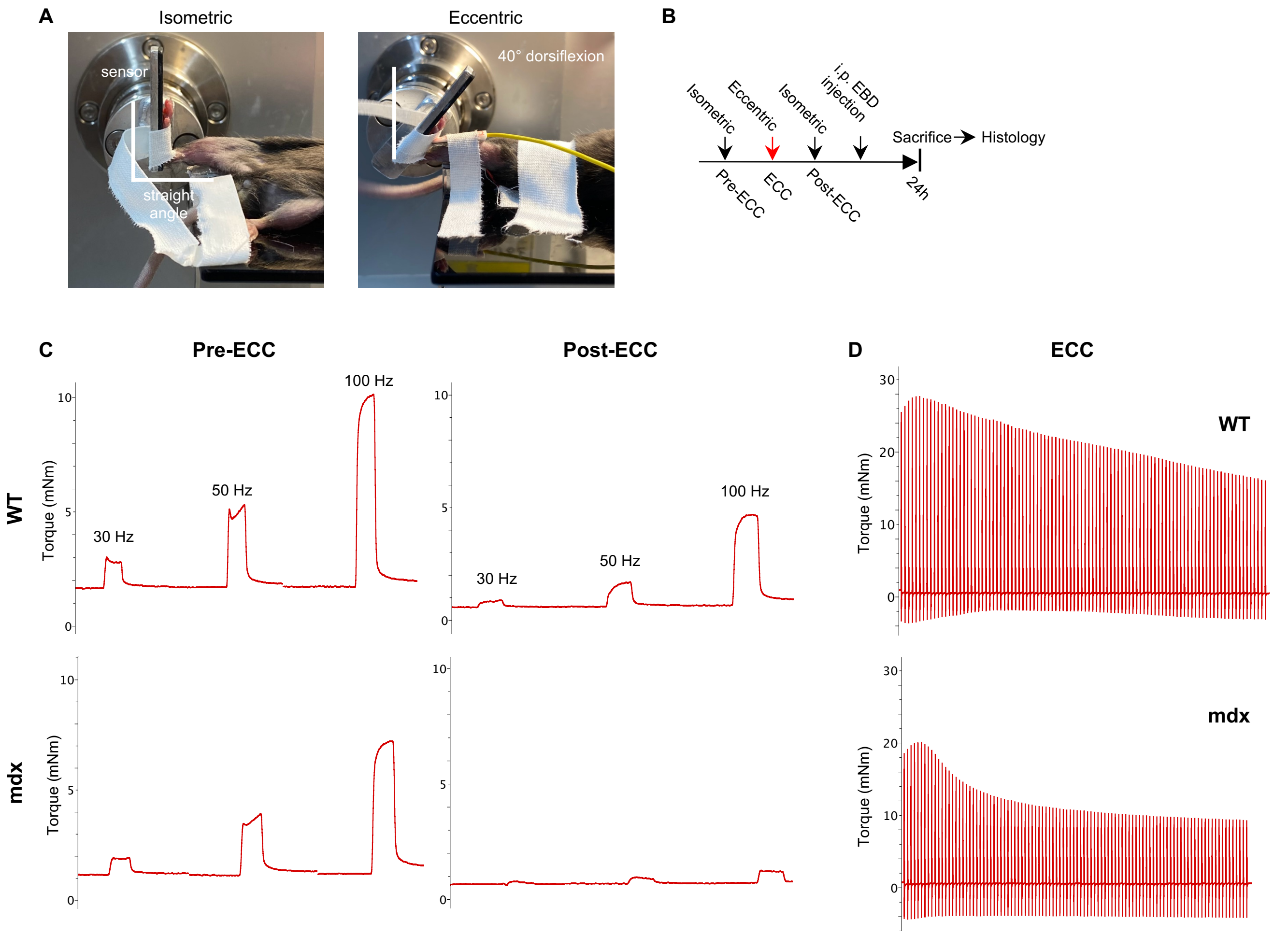

Figure S1. In vivo torque measurement technique for assessing muscle function in mice. (**A**) Photographs of the apparatus used for in vivo torque measurements, showcasing the setup for evaluating plantarflexor muscle function. (**B**) Schematic illustration of the protocol for measuring plantarflexor torque in vivo. (**C**) Representative isometric torque records of wild-type (WT) and mdx mice before and after the ECC session. (**D**) Representative eccentric torque records of WT and mdx mice.

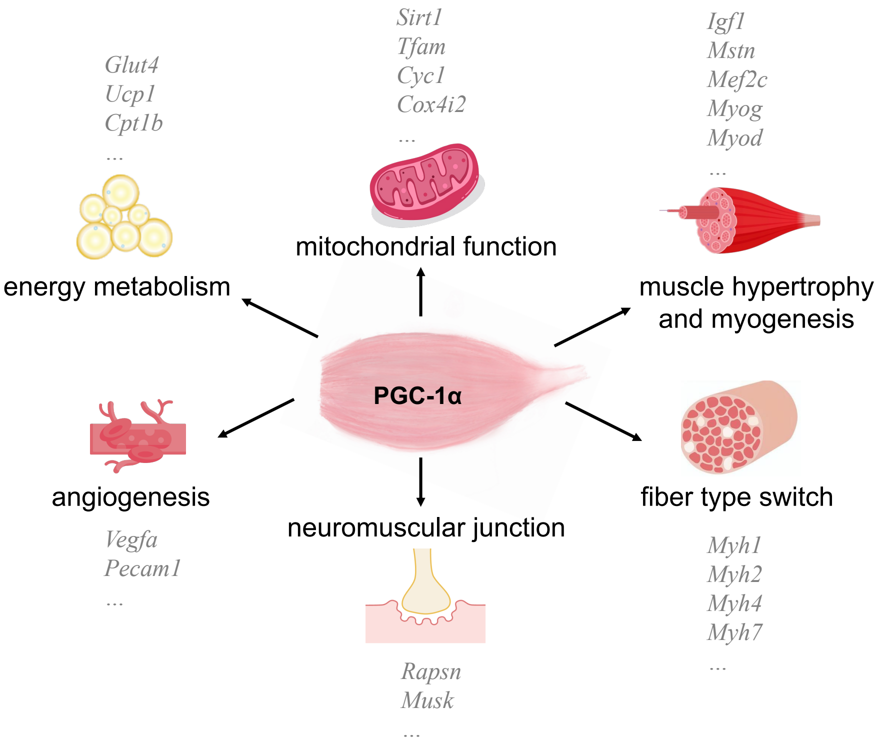

Figure S2. Putative PGC-1α interacting genes based on relevant literature (see citations [19-46, 62-68] in the main text).

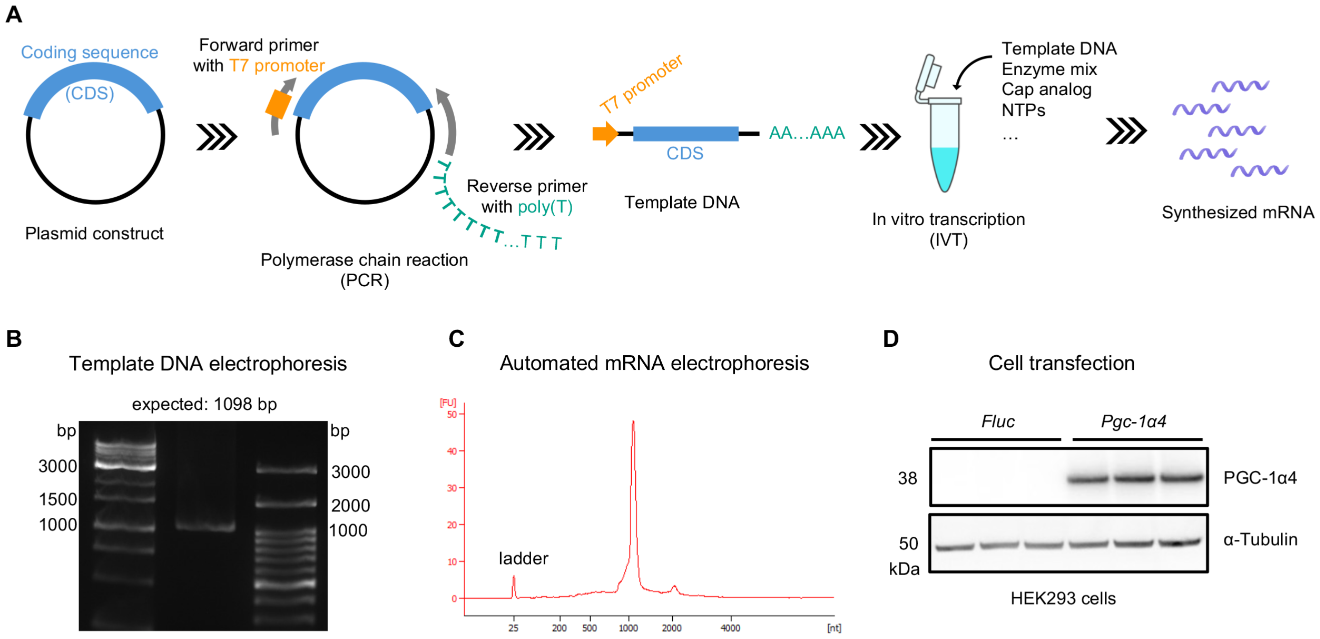

Figure S3. Preparation of mRNAs encoding PGC-1α4. (**A**) Overview of the mRNA synthesis process. (**B**) Representative gel electrophoresis image of template DNA used for in vitro transcription (IVT). (**C**) Automated mRNA electrophoresis data confirming the size and quality of the synthesized mRNA. Fluorescence units (FU) indicate sample intensity. (**D**) Western blot detection of PGC-1α4 following mRNA transfection into cells (n = 3 independent experiments). NTPs, nucleoside triphosphates.

5’ UTR | 3’ UTR | T7 promoter | Poly(A) tail

>5’UTR_Pgc-1α4_3’UTR

5’-GGCGAATTAAGAGAGAAAAGAAGAGTAAGAAGAAATATAAGACACCGGTCGCCACCATGCTGGGCCTGAGCAGCATG
GACAGCATCCTGAAGTGTGCTGCTCTCGTGGGCGAAGATCAGCCTCTGTGTCCTGATCTGCCTGAGCTGGACCTGTC
CGAGCTGGACGTGAACGACCTGGATACCGATAGCTTTCTCGGCGGCCTGAAGTGGTGTAGCGACCAGAGCGAGATC
ATCAGCAACCAGTACAACAACGAGCCCGCCAACATCTTCGAGAAGATCGACGAGGAAAACGAGGCCAACCTGCTGG
CCGTGCTGACAGAGACACTGGATAGCCTGCCTGTGGACGAGGATGGCCTGCCTTCTTTCGATGCCCTGACAGATGG
CGCCGTGACCACAGACAATGAGGCTAGCCCTAGCAGCATGCCTGACGGCACACCTCCACCTCAAGAGGCCGAAGAA
CCTAGCCTGCTGAAGAAGCTGCTGCTGGCCCCTGCTAACACCCAGCTGAGCTACAACGAGTGTAGCGGCCTGTCTA
CCCAGAACCACGCCGCTAACCACACACACAGAATCAGAACAAACCCCGCCATCGTCAAGACCGAGAACAGCTGGTCC
AACAAGGCCAAGAGCATTTGTCAGCAGCAGAAGCCCCAGAGAAGGCCCTGTTCTGAGCTGCTGAAGTACCTGACCAC
CAACGACGACCCTCCACACACCAAGCCTACCGAGAATAGAAACAGCAGCAGGGACAAGTGCGCCAGCAAGAAGAAG
TCTCACACCCAGCCTCAGAGCCAGCACGCTCAGGCTAAGCCTACCACACTGAGCCTGCCTCTGACACCTGAGAGCC
CCAACCTGTTCCTGTGAATCTAGACCTTCTGCGGGGCTTGCCTTCTGGCCATGCCCTTCTTCTCTCCCTTGCACCTGT
ACCTCTTGGTCTTTGAATAAAGCCTGAGTAGGAAAA -3’

>Forward primer

5’-CAGTGAATTGTAATACGACTCACTATAAGGCGAATTAAGAGAGAAAAGAAGAG -3’

>Reverse primer

5’-TTTTTTTTTTTTTTTTTTTTTTTTTTTTTTTTTTTTTTTTTTTTTTTTTTTTTTTTTTTTTTTTTTTTTTTTTTTTTTTTTTTTTTT
TTTTTTTTTTTTTTTTTTTTTTTTTTTTTTTTTCCTACTCAGGCTTTATTCA -3’

Figure S4. Codon-optimized sequences for 5’UTR_Pgc-1α4_3’UTR, and untranslated region (UTR)-specific primers.

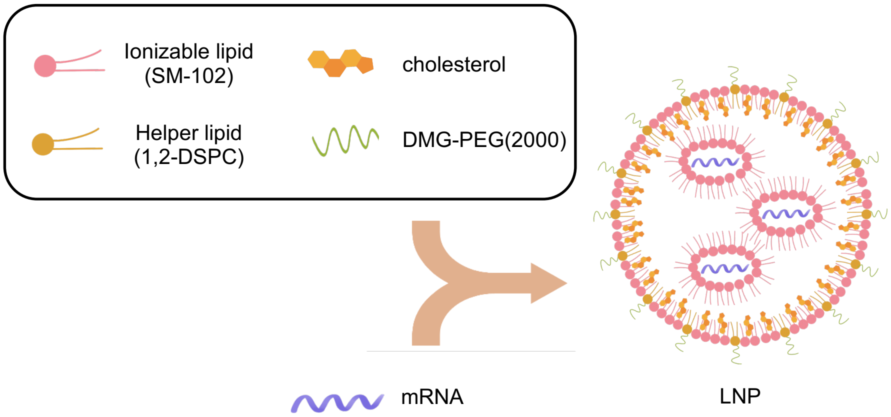

Figure S5. Schematic illustration of LNP formulation.

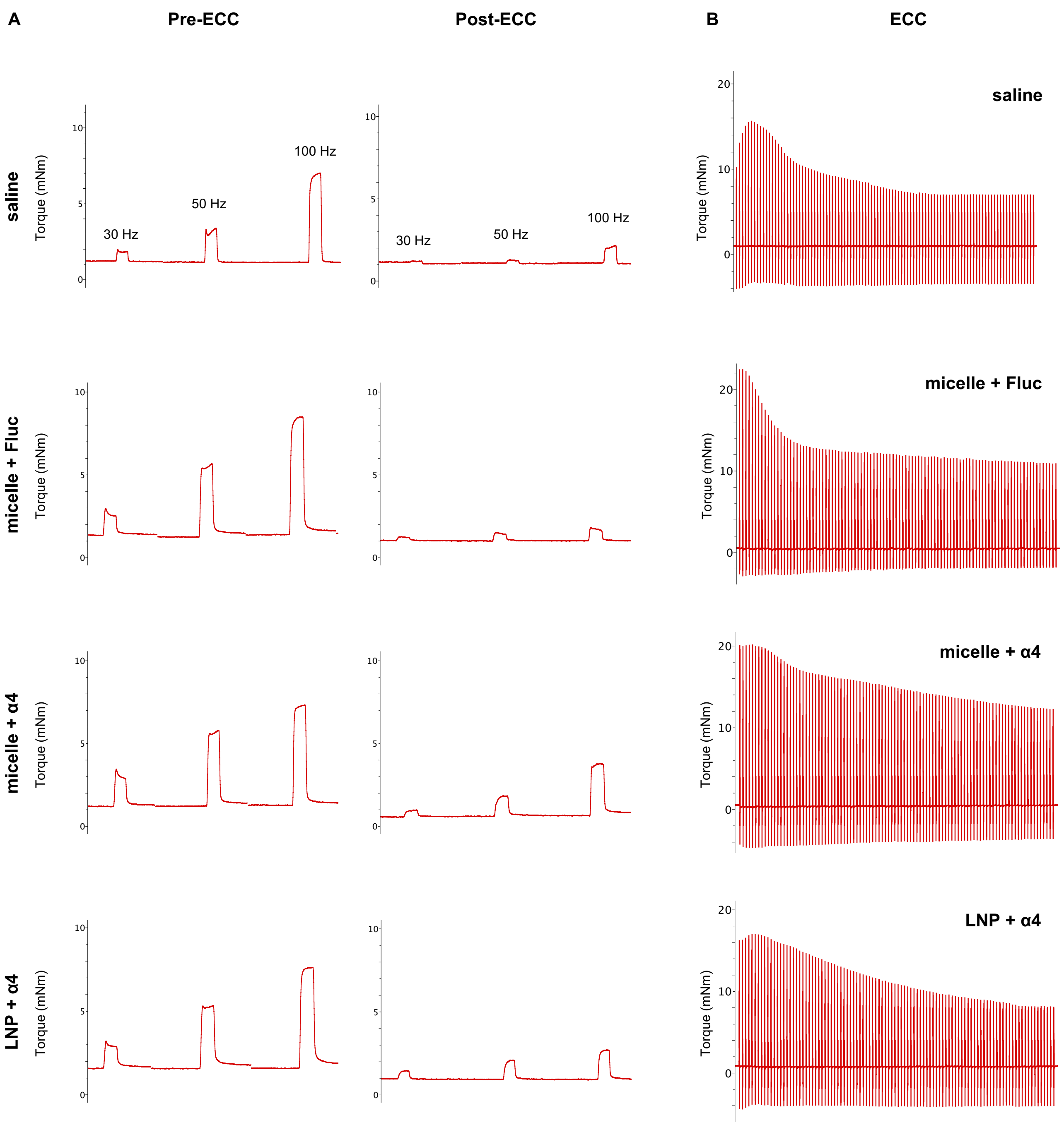

Figure S6. Representative isometric (**A**) and eccentric (**B**) torque records of mdx mice treated with saline, nanomielle-delivered *Fluc* mRNA, nanomielle-delivered *Pgc-1α4* mRNA, or LNP-delivered *Pgc-1α4* mRNA.

Table S1. The physicochemical properties of mRNA-loaded polyplex nanomicelles determined by dynamic light scattering (DLS).

	Polyplex nanomicelle	LNP
Size (nm)	56.8	121.6
Polydispersity (PDI)	0.127	0.078

Table S2. List of primers used for qPCR.

Gene	Forward primer	Reverse primer
Actb	GTGACGTTGACATCCGTAAAGA	GCCGGACTCATCGTACTCC
Total Pgc-1α	TGATGTGAATGACTTGGATACAGACA	GCTCATTGTTGTACTGGTTGGATATG
Pgc-1α1	GGACATGTGCAGCCAAGACTCT	CACTTCAATCCACCCAGAAAGCT
Pgc-1α4	CCTCACACCAAACCCACAGA	GGTCACTGGAAGATATGGCAC
Glut4	CCATCCTGATGACTGTGGCTCT	GCCACGATGAACCAAGGAATGG
Myod	GCACTACAGTGGCGACTCAGAT	TAGTAGGCGGTGTCGTAGCCAT
Mef2c	CGCAGGGAATGGATACGGCAAC	GGGATAAGAACGCGGAGATCTGG
Mstn	CAGACACACCCAAGAGGTCC	GGGTGCGATAATCCAGTCCC
Musk	CTGAAGGCTGTGAGTCCACTGT	TCCTTTACCGCCAGGCAGTACT
Pecam1	CCAAAGCCAGTAGCATCATGGTC	GGATGGTGAAGTTGGCTACAGG
Vegfa	CTGCTGTAACGATGAAGCCCTG	GCTGTAGGAAGCTCATCTCTCC
Myog	CCATCCAGTACATTGAGCGCCT	CTGTGGGAGTTGCATTCACTGG
Fndc5	GATGTCCTGGAGGATGAAGTGG	GTGGTGGTGTTCACCTCCTGAA
Bdnf	GGCTGACACTTTTGAGCACGTC	CTCCAAAGGCACTTGACTGCTG
Cpt1b	ATGTATCGCCGCAAACTGGACC	CTCTGAGAGGTGCTGTAGCAAG
Cox4i2	CTGCCCGGAGTCTGGTAATG	CAGTCAACGTAGGGGGTCATC
Cyc1	CCATCTACACAGAAGTCTTGGAG	GCGTTTTCGATGGTCATGCTCTG
Tfam	ATTCCGAAGTGTTTTTCCAGCA	TCTGAAAGTTTTGCATCTGGGT
Rapsn	ATATCGGGCCATGAGCCAGTAC	TCACAACACTCCATGGCACTGC
Igf1	GTGGATGCTCTTCAGTTCGTGTG	TCCAGTCTCCTCAGATCACAGC
Sirt1	GACGCTGTGGCAGATTGTTA	GGAATCCCACAGGAGACAGA
Myh2	GGAGGCTGAGGAACAATCCA	GCATCGGGACAGCCTTACTC
Myh7	GAATGGCAAGACGGTGACTGTG	GGAAGCGTAGCGCTCCTTGAG
Myh4	ACAGACTAAAGTGAAAGCCTACAA	CACATTTTGTGATTTCTCCTGTCAC
Myh1	GCATCCCTAAAGGCAGGCTC	CAAACACCGATGACTTGGCG
Ucp1	ACTGCCACACCTCCAGTCATT	CTTTGCCTCACTCAGGATTGG
Mcp1	AGCTGTAGTTTTTGTCACCAAGC	GTGCTGAAGACCTTAGGGCA
